# Acoustic Trauma Modulates Cochlear Blood Flow and Vasoactive Factors in a Rodent Model of Noise-Induced Hearing Loss

**DOI:** 10.3390/ijms20215316

**Published:** 2019-10-25

**Authors:** Sun-Ae Shin, Ah-Ra Lyu, Seong-Hun Jeong, Tae Hwan Kim, Min Jung Park, Yong-Ho Park

**Affiliations:** 1 Department of Otolaryngology-Head and Neck Surgery, College of Medicine, Chungnam National University, Daejeon 35015, Korea; ttd0707@naver.com (S.-A.S.); ahmilove@naver.com (A.-R.L.); 2 Department of Medical Science, College of Medicine, Chungnam National University, Daejeon 35015, Korea; hunpass2@gmail.com; 3 Biomedical Convergence Research Center, Chungnam National University Hospital, Daejeon 35015, Korea; czkth@naver.com; 4 Brain Research Institute, College of Medicine, Chungnam National University, Daejeon 35015, Korea

**Keywords:** hearing loss, noise-induced, regional blood flow, stria vascularis

## Abstract

Noise exposure affects the organ of Corti and the lateral wall of the cochlea, including the stria vascularis and spiral ligament. Although the inner ear vasculature and spiral ligament fibrocytes in the lateral wall consist of a significant proportion of cells in the cochlea, relatively little is known regarding their functional significance. In this study, 6-week-old male C57BL/6 mice were exposed to noise trauma to induce transient hearing threshold shift (TTS) or permanent hearing threshold shift (PTS). Compared to mice with TTS, mice with PTS exhibited lower cochlear blood flow and lower vessel diameter in the stria vascularis, accompanied by reduced expression levels of genes involved in vasodilation and increased expression levels of genes related to vasoconstriction. Ultrastructural analyses by transmission electron microscopy revealed that the stria vascularis and spiral ligament fibrocytes were more damaged by PTS than by TTS. Moreover, mice with PTS expressed significantly higher levels of proinflammatory cytokines in the cochlea (e.g., IL-1β, IL-6, and TNF-α). Overall, our findings suggest that cochlear microcirculation and lateral wall pathologies are differentially modulated by the severity of acoustic trauma and are associated with changes in vasoactive factors and inflammatory responses in the cochlea.

## 1. Introduction

The World Health Organization estimates that 12% or more of the global population is at risk of hearing loss from noise, which impacts more than 600 million people worldwide [1,2,3]. The annual cost of hearing impairment is within the range of $750 to $790 billion globally [4]. Noise-induced hearing loss (NIHL) occurs with single or repeated sudden noise exposure and is a major health problem [2,5,6,7]. Noise exposure results in a wide range of cochlear damage including blood-flow reduction and capillary constriction, as well as changes in microcirculation, lateral wall, and spiral ligament fibrocytes (SLFs) [8,9,10,11,12,13].

A significant part of cells within the inner ear consists of connective tissue cells of the spiral ligament, but relatively little is known regarding their functional significance. The cochlear lateral wall, including the spiral ligaments and stria vascularis, is an area that is strongly affected by noise, due to its role in the maintenance of cochlear fluid homeostasis [13]. The major capillary systems in the spiral ligament and stria vascularis form four distinct networks (supra- and post-strial capillary networks of the spiral ligament, ad-strial capillary network of the spiral ligament, and capillaries of the stria vascularis) [14] which modulate cochlear endolymph homeostasis through generation of ionic gradients and K^+^ recycling between perilymph and endolymph [15]. These capillaries, therefore, play crucial roles in controlling sensory hair cell transduction by regulating endocochlear potential, ion transport, and endolymphatic fluid balance [16,17,18,19,20]. Dysfunction of the cochlear lateral wall is considered a potential etiology for a number of hearing disorders, including NIHL [10,21,22,23].

Functional hearing recovery is strongly associated with morphological remodeling of the cochlear lateral wall and repair of the SLFs [24,25]. There is increasing evidence that SLFs have a remarkably low threshold for noise-induced loss, which may explain the prevalence of missing fibrocytes in humans [26]. Loss of fibrocytes reportedly begins at a young age and progressively increases with time, according to clinical studies [26,27,28]. Moreover, the earliest change in aging ears is found in fibrocytes, rather than in hair cells or neurons [29,30]. Fibrocytes play a key role in K^+^ recycling to the stria vascularis and endolymph from the organ of Corti [31]. Changes in SLFs are accompanied by hearing loss and a large reduction in endolymphatic potential [32].

The main targets of noise-induced damage have been extensively studied in hair cells, spiral ganglion neurons (SGNs), and nerve fibers, however, much less is known regarding damage to the cochlear lateral wall. Thus, we investigated how the spiral ligament and microcirculation in the lateral wall are modulated after different severities of acoustic trauma. The physiological characteristics of cochlear blood flow and spiral ligament fibrocytes after noise exposure were determined by measuring changes in vasoactive factors and inflammatory and oxidative stress responses.

## 2. Results

### 2.1. Auditory Brainstem Response Threshold by Transient Hearing Threshold Shift and Permanent Hearing Threshold Shift

**Figure 1:**
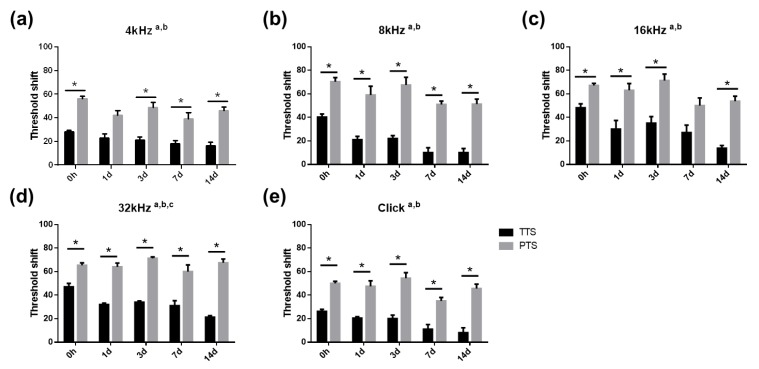
Auditory brainstem response (ABR) threshold shifts after noise exposure. ABR thresholds were measured at the following six time points: prior to (pre), immediately after (0 h), 1 d, 3 d, 7 d, and 14 d after noise exposure and graphed for ABR threshold shifts. Permanent hearing threshold shift (PTS) group showed significantly increased ABR threshold shift as compared with transient hearing threshold shift (TTS) animals at all frequencies and click stimuli at all-time points (**a**–**e**). TTS, transient threshold shift; PTS, permanent threshold shift; a, main effect of day, *p* < 0.05; b, main effect of treatment, *p* < 0.05; and c, interaction, *p* < 0.05. All graphs represent mean ± S.E.M. Two-way ANOVA, Tukey’s multiple comparisons test. * *p* < 0.05, *n* = 25, 0 h; *n* = 5, 1 d, 3 d, and 7 d; *n* = 4 and 14 d.

To evaluate how noise exposure transient hearing threshold shift (TTS) or permanent hearing threshold shift (PTS) changes the hearing threshold in mice, auditory brainstem response (ABR) thresholds at 4, 8, 16, and 32 kHz, and for click sounds were measured at the following six time points: before noise exposure (baseline or pre); immediately after (0 h); and at one, three, seven, and 14 days after noise exposure. As shown in Figure 1a–e, for all frequencies and click sound stimuli, mice with PTS had a significantly greater ABR threshold shift as compared to mice with TTS (a, main effect of day, *p* < 0.05; b, main effect of treatment, *p* < 0.05 for all frequencies and click sounds, two-way ANOVA, Tukey’s multiple comparisons test). The ABR threshold shift significantly increased immediately after noise exposure in both TTS and PTS mice. Mice with TTS showed a gradual reduction in threshold shift, while those with PTS maintained a high level of threshold shift until 14 days after noise exposure, which indicates that TTS mice recovered from noise trauma over time, whereas PTS mice did not.

### 2.2. Survival of SGNs is Modulated by Severity of Noise Trauma

**Figure 2:**
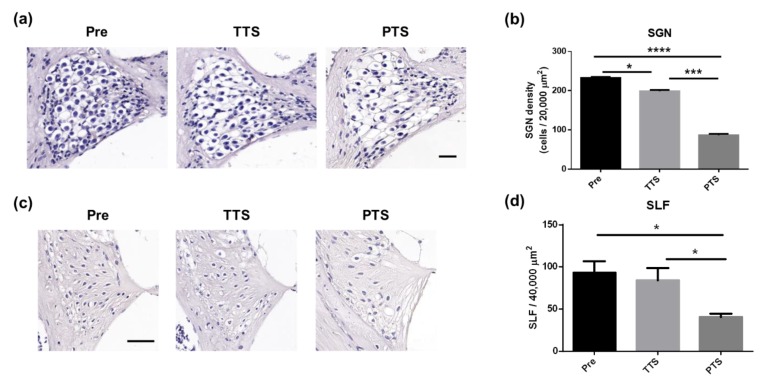
Changes of spiral ganglion neurons (SGNs) and spiral ligament after noise exposure. (**a**) SGNs were observed before (pre) and 14 d after (post) noise exposure. Representative pictures (**a**) and the number of SGNs (**b**) in the basal turn were obtained. (**b**) Both TTS and PTS groups showed a significant reduction in SGNs (hematoxylin positive cells/20,000 μm^2^) after noise exposure. PTS induced a further decrease in SGN as compared to TTS animals at 14 d post noise trauma. Scale bars represent 20 μm. (**c**) A major cell type in spiral ligament, type IV fibrocytes, were visualized by nucleic acid staining (Hematoxylin) before (normal) and 14 d after noise exposure. (**d**) The number of spiral ligament type IV fibrocytes (SLFs) in the basal turns of cochlea was significantly decreased by PTS at 14 d post noise exposure. Scale bar represents 50 μm. Graphs represent mean ± S.E.M. *n* = 4. One-way ANOVA with Tukey’s multiple comparisons test. *p < 0.05, *** *p* = 0.003, and **** *p* < 0.0001.

SGNs in the basal turn were visualized by hematoxylin staining. The neurons were quantified before (pre) and 14 days after noise exposure. As shown in Figure 2, there were significant reductions in neuronal density (hematoxylin-positive cells/20,000 μm^2^) in both groups, mice with TTS (* *p* = 0.01) and mice with PTS (**** *p* < 0.0001) after acoustic overstimulation (two-way ANOVA, Tukey’s multiple comparisons test). Mice with PTS exhibited a further reduction in SGN density as compared to mice with TTS, at 14 days (*** *p* = 0.003) after noise trauma. These data indicate that SGNs experienced more damage in PTS than in TTS mice.

### 2.3. Changes in Spiral Ligament Fibrocytes

Changes in fibrocytes around the spiral ligament in the lateral wall were evaluated before and at 14 days after noise exposure. Representative images of SLFs were obtained (Figure 2c) and quantified (Figure 2d). The number of SLFs in the basal turn of the cochlea at 14 days after noise exposure were significantly reduced by PTS, whereas the number of SLFs were not reduced by TTS after acoustic trauma (*p* > 0.05, pre vs. TTS); this indicates that SLFs experienced greater impairment in the PTS mice than in the TTS mice.

**Figure 3:**
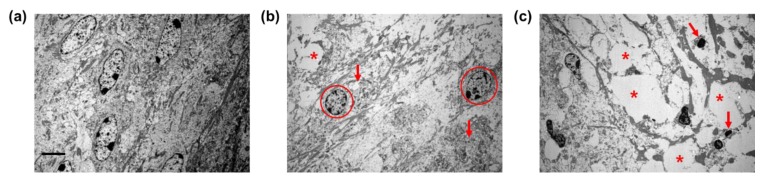
Ultrastructural analysis of spiral ligament type IV fibrocytes before (normal control) and 14 d after TTS or PTS. (**a**) Normal spiral ligament presented cylindrical, spindle-shaped type IV fibrocytes. (**b**) Type IV fibrocytes at 7 d after TTS showed swollen spherical nuclei (circles), cellular debris (red arrows), and vacuolization (asterisk). (**c**) PTS induced massive cytoplasmic vacuolization (asterisk) in the type IV fibrocytes and displayed several apoptotic bodies (red arrows). Scale bar represents 2 μm. *n* = 3.

Ultrastructural analyses of the spiral ligament architecture revealed the presence of fusiform type-IV fibrocytes containing granular cytoplasm (Figure 3a). The spiral ligament degenerated in TTS mice, as indicated by rounded nuclei and vacuolization (Figure 3b). Compared to TTS mice, the PTS mice exhibited more widespread cell loss with vacuolization; apoptotic cell bodies; and condensed, dark, and shrunken nuclei (Figure 3c). These data suggest that the spiral ligament was affected by noise exposure and that the damage was more severe in PTS mice.

### 2.4. Acoustic Trauma Modulates Cochlear Microcirculation

**Figure 4:**
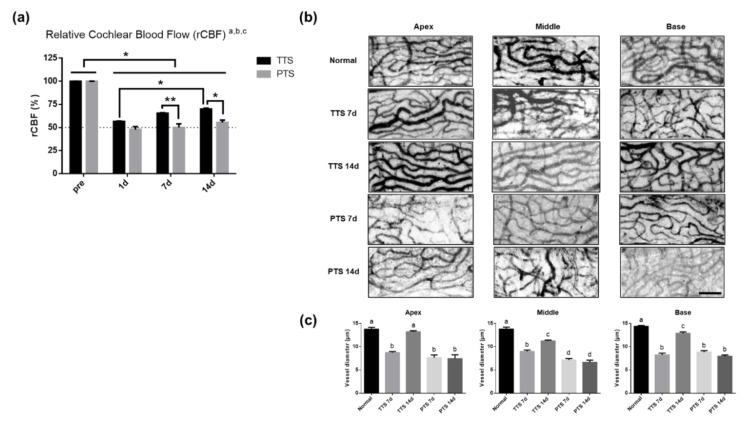
Changes of cochlear blood flow and microvasculature of stria vascularis after noise exposure. (**a**) The cochlear blood flow was measured in TTS and PTS groups pre and post (1 d, 7 d, and 14 d) noise exposure. Both TTS and PTS significantly decreased cochlear blood flow at 1 d post noise exposure. The blood flow significantly increased in the TTS group as compared with the PTS group at 7 d and 14 d post noise exposure. TTS, transient threshold shift; PTS, permanent threshold shift; a, main effect of day, *p* < 0.05; b, main effect of treatment, *p* < 0.05. c, interaction, *p* < 0.05; *p < 0.05, ** *p* < 0.01. All graphs represent mean ± S.E.M. Two-way ANOVA, Tukey’s multiple comparisons test. (**b**) Endothelial cells of stria vascularis were stained with anti-PECAM antibody, and confocal images were obtained before (normal) and after (7 d and 14 d) noise exposure (TTS or PTS). Representative pictures of confocal images. Scale bar represents 100 μm. (**c**) Capillary thickness was measured in diameter and quantified. The vessel diameter in apex was significantly decreased by PTS at 7 d and 14 d as compared with normal animals, while that of TTS recovered toward normal thickness at 14 d. All graphs represent mean ± S.E.M. One-way ANOVA, Tukey’s multiple comparisons test. a vs. b vs. c, *p* < 0.05. *n* = 12, normal; *n* = 6, all other groups.

To evaluate whether the severity of noise trauma differentially affects cochlear microvasculature, blood flow was measured before (pre) and after (at one, seven, and 14 days) noise exposure with a laser Doppler flowmeter. Cochlear blood flow was significantly reduced at all measured time points in both groups as compared to the baseline (Figure 4, pre vs. all other time points, *p* < 0.05, Tukey’s multiple comparisons test). The TTS mice showed a significant reduction one day after trauma (*p* < 0.0001, pre vs. TTS at 1 day) and a restored pattern over time up to 14 days (*p* = 0.0195, TTS at one day vs. TTS at 14 days). Importantly, blood flow in PTS mice did not recover by 14 days after noise exposure (*p* < 0.05, pre vs. PTS at one, seven, and 14 days). Mice with PTS exhibited significantly lower cochlear blood flow at 7 days (*p* = 0.0086, TTS vs. PTS) and 14 days (*p* = 0.0129, TTS vs. PTS) after noise trauma as compared with TTS mice. These findings imply that noise exposure was associated with cochlear blood flow reduction, leading to cochlear damage or ischemia, and that the severity of noise trauma was negatively correlated with the speed of threshold recovery.

### 2.5. Microvasculature of Stria Vascularis after Acoustic injury

Next, we tested whether a threshold shift modulates the vessel diameter of the cochlear lateral wall. Micro-dissected lateral wall tissues (apex, middle, and base) were stained with anti-PECAM1 antibody before (normal) and after (at seven and 14 days) noise exposure; they were imaged (Figure 4b) and quantified (Figure 4c). As shown in Figure 4c, the vessel diameter of the cochlear lateral wall was significantly reduced at seven days after noise trauma in TTS and PTS mice in apex tissues (one-way ANOVA, Tukey’s multiple comparisons test, *p* < 0.0001, pre vs. TTS at seven days apex), middle tissues (*p* < 0.0001, pre vs. TTS at seven days middle) and base tissues (*p* < 0.0001, pre vs. TTS, at seven at days base). Mice with TTS exhibited restored vessel diameter in the apex at 14 days after acoustic trauma (*p* = 0.9232, normal vs. TTS at 14 days; *p* < 0.0001, TTS at seven days vs. TTS at 14 days), while mice with PTS exhibited a persistently reduced diameter by 14 days (*p* = 0.9991, PTS at seven days vs. PTS at 14 days; *p* < 0.0001, normal vs. PTS at seven and 14 days).

**Figure 5:**
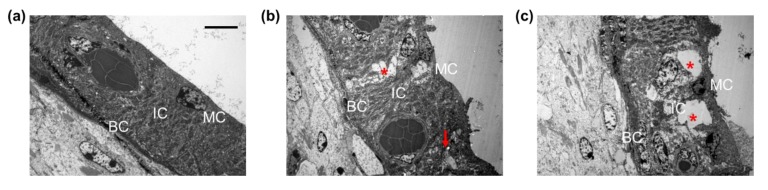
Ultrastructural analysis of stria vascularis before (normal control) and after (14 d) TTS or PTS. (**a**) Normal stria vascularis showed intact three layers of strial cells. (**b**) TTS-treated stria vascularis exerts small vacuolization (asterisk) or gaps (arrow) between the strial cells. (**c**) PTS-induced stria vascularis contained the large vacuolization (asterisk) between the cellular processes of strial cells. Scale bar represents 5 μm. BC, basal cells; IC, intermediate cells; and MC, marginal cells. *n* = 3.

The stria vascularis (Figure 5a) of normal mice has three cell types, basal, intermediate, and marginal [13,33,34], but in ultrastructural analyses in our study, its structure was unclear and vacuolated in both mice groups (Figure 5b–c). Furthermore, the stria vascularis had larger gaps between the cellular processes of strial cells in mice with PTS than in mice with TTS. These data imply that stria vascularis architecture in the cochlear lateral wall was affected by acoustic trauma and that the vessel diameter was differentially modulated by the severity of hearing threshold shifts.

### 2.6. Stria Vascularis Thickness is Changed by Noise Exposure

**Figure 6:**
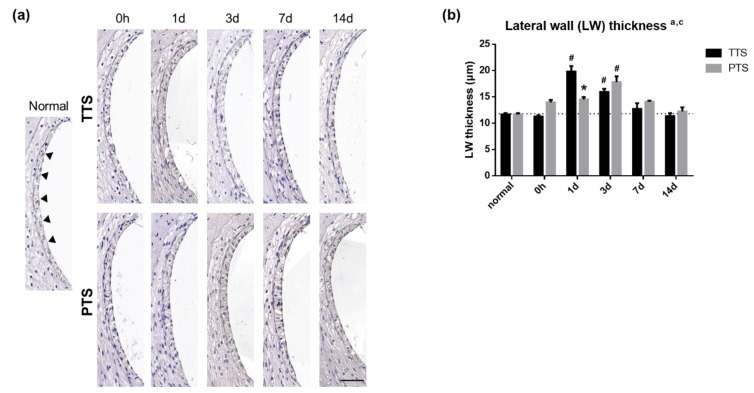
Thickness of lateral wall after noise exposure. (**a**) Hematoxylin staining was performed on inner ear section at each time point. (**b**) After noise trauma, the stria vascularis thickness was significantly increased in TTS and PTS groups. Scale bars represent 50 μm. a, main effect of day, *p* < 0.05 and c, interaction, *p* < 0.05. All graphs represent mean ± S.E.M. Two-way ANOVA, Tukey’s multiple comparisons test. Asterisk denotes difference from TTS per day, *p* < 0.05 and pound symbols (#) denote differences from matching normal (pre) controls, *p* < 0.05. *n* = 3.

To investigate how noise trauma affects lateral wall thickness, hematoxylin staining was performed on inner-ear sections before (normal) and after (immediately, and one, three, seven, and 14 days) noise exposure; it was quantified using a two-way ANOVA with a Tukey’s multiple comparisons test (a, main effect of day, *p* < 0.05; c, interaction, *p* < 0.05). Stria vascularis thickness was significantly increased at one day (# *p* < 0.0001) and three days (# *p* = 0.0013) after acoustic trauma in mice with TTS as compared with those same mice prior to the induction of TTS (Figure 6). Mice with TTS exhibited significantly swollen stria vascularis at one day after noise trauma and a full recovery to normal thickness by 14 days after noise trauma, while the stria vascularis in mice with PTS exhibited peak thickness at three days after noise exposure. Stria vascularis thickness was fully recovered by seven days after noise trauma in both TTS and PTS mice.

### 2.7. Vasoactive Genes are Differentially Expressed in Both TTS and PTS

**Figure 7:**
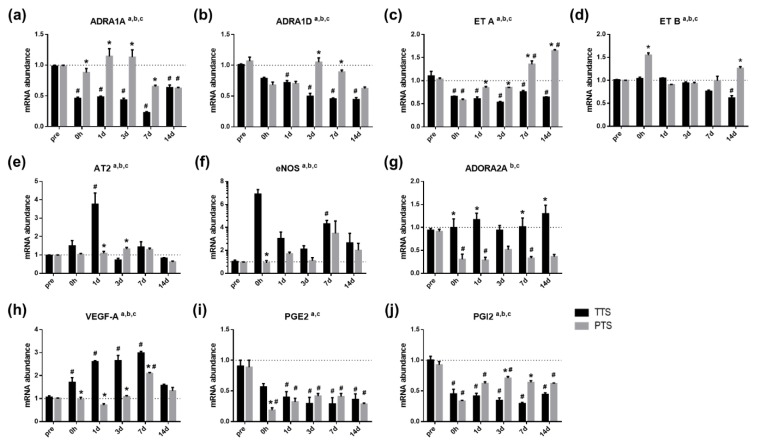
Genes involved in vasoconstriction and in vasodilation before (pre) and after (post) noise exposure. Effects of noise on vascular function, sensitivity to vasoconstriction (**a**–**d**) and vasodilation (**e**–**j**). a, main effect of day, *p* < 0.05; b, main effect of treatment (TTS, PTS), *p* < 0.05; and c, interaction, *p* < 0.05; *p < 0.05. All graphs represent mean ± S.E.M. Two-way ANOVA, Tukey’s multiple comparisons test. a, main effect of day, *p* < 0.05; b, main effect of treatment, *p* < 0.05; and c, interaction, *p* < 0.05. Asterisk denotes difference from TTS per day, *p* < 0.05; pound symbols (#) denote differences from matching normal (pre) controls, *p* < 0.05. *n* = 3–5.

Gene expression levels associated with vasoconstriction and vasodilation (Figure 7) were measured before (pre) and after (0 h, and one, three, seven, and 14 days) noise exposure. Genes involved in vasoconstriction included alpha-1A adrenergic receptor (*ADRA1A*) [35], alpha-1D adrenergic receptor (*ADRA1D*) [36,37], endothelin receptor type A (*ET A*) [38,39], and endothelin receptor type B (*ET B*) [38]. All “vasoconstrictive” genes exhibited an interaction effect (*p* < 0.05, two-way ANOVA) and main effect of both treatment (TTS vs. PTS) and time (*p* < 0.05, two-way ANOVA), indicating that genes involved in vasoconstriction were differentially modulated by the intensity of noise (b, main effect of treatment, *p* < 0.05). Corrected comparisons (Tukey’s multiple comparison test) between the groups at each time point revealed that mice with PTS showed significantly higher “vasoconstrictive” gene expression after noise trauma as compared to mice with TTS (Figure 7, *p* < 0.05, two-way ANOVA, Tukey’s multiple comparisons test). Noticeably, all “vasoconstrictive” genes at all time points showed similar or lower expression levels in both TTS and PTS mice as compared twith normal controls; furthermore, only the levels of ET A were higher in PTS mice at seven and 14 days as compared with normal controls. (#, differences from matching normal controls, *p* < 0.05).

Genes involved in vasodilation included angiotensin 2 receptor type 2 (*AT2*) [40,41,42], endothelial nitric oxide synthase (*eNOS*) [43], adenosine A2A receptor (*ADORA2A*) [44,45], *VEGF-A* [43], prostaglandin E receptor 2 (*PGE2*) [46], and prostaglandin I2 receptor (*PGI2*) [47]. The expression levels of *AT2* (Figure 7e), *eNOS* (Figure 7f), *VEGF-A* (Figure 7h), and *PGI2* (Figure 7i) exhibited interaction effects (*p* < 0.05, two-way ANOVA) and a main effect for both time and treatment (TTS vs. PTS, *p* < 0.05, two-way ANOVA). Planned comparisons (Tukey’s multiple comparisons test) between groups at each time point revealed that mice with TTS exhibited significantly higher expression levels of “vasodilating” genes after acoustic trauma as compared to mice with PTS (Figure 7, two-way ANOVA, Tukey’s multiple comparisons test). Increased expression levels of *AT2, eNOS*, and *VEGF-A* were observed in mice with TTS as compared with normal controls (Figure 7e,f,h). Other “vasodilating” genes, such as *ADORA2A* and *PGE2*, were increased in mice with TTS as compared to mice with PTS (Figure 7g,i). Overall, expression levels of “vasodilating” genes were significantly higher in mice with TTS than in mice with PTS. These data suggest that genes involved in vasoconstriction and vasodilation are differentially modulated by the severity of noise trauma and may impact lateral wall pathology, microvessel diameter, and cochlear blood flow.

### 2.8. Genes Involved in Oxidative Stress are Modulated by Noise Trauma

**Figure 8:**
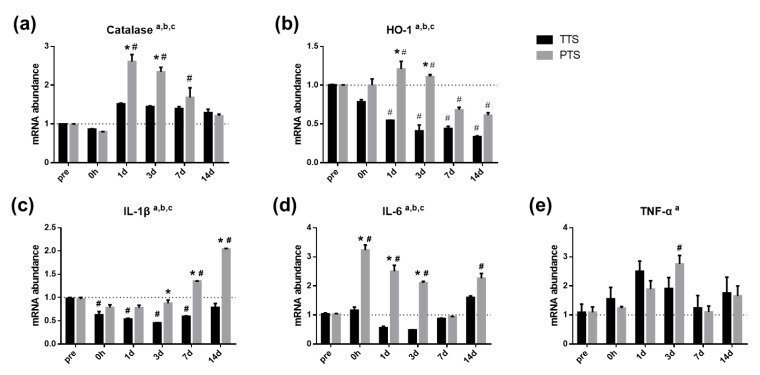
Oxidative stress responses and proinflammatory cytokines before and after noise exposure. The qRT-PCR for antioxidant enzymatic scavengers (**a**–**b**) and inflammatory cytokines (**c**–**e**) before (pre) and after (0 h, 1 d, 3 d, 7 d, and 14 d) noise trauma (TTS and PTS). a, main effect of day, *p* < 0.05; b, main effect of treatment (TTS, PTS), *p* < 0.05; c, interaction, *p* < 0.05; * *p* < 0.05. All graphs represent mean ± S.E.M. Two-way ANOVA, Tukey’s multiple comparisons test. Asterisk denotes difference from TTS per day, *p* < 0.05 and pound symbols (#) denote differences from matching normal (pre) controls, *p* < 0.05. *n* = 3–5.

To determine how severity of noise trauma differentially affects oxidative stress responses in the cochlea, quantitative real-time polymerase chain reaction assays for catalase [48,49] and heme oxygenase 1 (*HO-1*) [48] were conducted before (pre) and after (0 h, and one, three, seven, and 14 days) acoustic trauma. As shown in Figure 8a,b, both catalase and *HO-1* exhibited a main effect of treatment (TTS vs. PTS, *p* < 0.0001, two-way ANOVA) and main effect of time (*p* < 0.0001, two-way ANOVA). Catalase was significantly increased in mice with PTS at one, three, and seven days after noise trauma as compared with the pre-PTS level (*p* < 0.05, pre vs. PTS at one, three, and seven days, hash symbols). Planned comparisons revealed that mice with PTS exhibited significantly higher catalase expression as compared to mice with TTS, at one and three days after noise exposure. *HO-1* expression was also upregulated in mice with PTS as compared to mice with TTS, at one and three days after acoustic trauma. These data suggest that oxidative stress responses are differentially modulated by noise trauma of different severities, which may affect vessel pathology.

### 2.9. Genes Involved in Proinflammatory Responses are Modulated by Noise Trauma

To test cytokine levels in the cochlea, tissue samples were collected from mice with TTS or PTS before (pre) and after (0 h, and at one, three, seven, and 14 days) noise trauma. Quantitative real-time polymerase chain reaction assays were performed to examine the levels of interleukin-1β (*IL-1β*), interleukin-6 (*IL-6*), and tumor necrosis factor-α (*TNF-α*). Figure 8c–e shows that the expression levels of *IL-1β*, *IL-6*, and *TNF-α* were significantly modulated by noise trauma (a, main effect of day, *p* < 0.05; b, main effect of treatment (TTS, PTS), *p* < 0.05; and c, interaction, *p* < 0.05). The *IL-1β* expression levels were significantly increased in mice with PTS as compared to mice with TTS, at later time points (three, seven, and 14 days), whereas *IL-6* expression levels were increased at earlier time points (immediately, and at one and three days) after noise trauma. *TNF-α* exhibited a main effect of time, but not of treatment. Overall, mice with PTS exhibited significantly higher expression levels of proinflammatory cytokines as compared to mice with TTS (Figure 8c–e). These observations suggest that noise exposure induced local inflammation was more severe in the PTS group.

## 3. Discussion

In this study, we evaluated how the cochlear lateral wall is affected by different noise conditions. Noise exposure induced structural changes in the cochlea, including a loss of spiral ganglion neurons (Figure 2a,b) and structural changes in spiral ligament type IV fibrocytes (Figure 2) and the stria vascularis (Figure 6). Acoustic overstimulation adversely affected cochlear microcirculation by causing blood vessel contraction (Figure 4b,c), which led to reduced blood flow (Figure 4a). Mice with PTS exhibited more severe damage than mice with TTS. A significant increase in “vasoconstrictive” genes (Figure 7a–d) and a significant decrease in vasodilating genes (Figure 7e–j) were observed in PTS mice. Blood flow reduction caused by PTS induced local ischemic damage and subsequent cochlear inflammation (Figure 8c–e) and these findings are consistent with prior studies [50,51].

Cochlear blood flow and oxygen levels decline during noise exposure, consistent with the general concept of ischemia and reperfusion as an important pathophysiological process in NIHL [8,52,53,54,55,56]. Noise exposure results in multiple impairments of cochlear microcirculation, including increased vascular permeability and reduced cochlear blood circulation [21,22]. Although some research has reported reductions in blood supply in response to noise trauma, few studies have confirmed the original findings [51]. For instance, Okamoto et al. observed no changes in guinea pig cochlear blood flow in response to 120 dB sound pressure level (SPL) [57]. Hultcrantz was unable to show a significant alteration in cat cochlear blood flow by 100 dB noise for 6 min [58]. Prazma et al. also found no significant change in cochlear blood flow by 115 dB noise for 6 h [59].

More recent findings have corroborated the changes in cochlear blood flow after loud noise, due to technical developments such as intravital microscopy. These studies have demonstrated that stria vascularis vessels exhibit an initial compensatory increase in red blood cell velocity within 30 to 180 s after noise exposure, followed by a reduction in blood flow after several minutes [26,51,60]. Surprisingly, after 30 min of noise exposure, the flow was reversed and stasis ultimately occurred in many of the capillaries within the stria vascularis [26,51,60]. Arpornchayanon et al. also reported a reduction in guinea pig cochlear blood flow when measured at 106 dB SPL with a duration of 30 min [10]. Other investigators have reported that alterations in cochlear blood flow and hypoxia are strictly correlated with the intensity of noise and severity of hearing loss [8,55]. Our data also support changes in cochlear blood flow in response to hearing threshold shifts (Figure 4a). Cochlear blood flow in mice with TTS was restored by 14 days after noise exposure, whereas that in mice with PTS was not, suggesting that cochlear blood flow reduction and recovery are associated with the severity of noise trauma.

On the basis of the results shown in Figure 4a, it is important to determine how vessel tone is modulated. When we measured vasoactive factors (Figure 7) in cochlear homogenates, mice with PTS exhibited significantly higher expression levels of genes involved in vasoconstriction (e.g., endothelin receptors) and lower expression levels of “vasodilating” genes (e.g., vascular endothelial growth factor [VEGF] and adenosine A2A receptors) as compared to mice with TTS (Figure 7). Importantly, genes involved in vasoconstriction and vasodilation were differentially modulated by the intensity of noise (b, main effect of treatment, *p* < 0.05). All “vasoconstrictive” genes (Figure 7a–d) at all time points showed similar or lower expression levels in both TTS and PTS mice as compared with those in normal controls. Conversely, endothelin (ET) A showed an increased level only in mice with PTS at seven days and 14 days as compared with normal controls (#, differences from matching normal controls, *p* < 0.05). ET, a potent vasoconstrictor peptide, functions as a local hormonal regulator of neurotransmitters, ions, and pressure in the inner ear [61] by binding to the receptors ET A and ET B [62]. Both of these are expressed in the strial vascularis and in non-strial tissues. Moreover, capillary constriction in the spiral ligament is regulated by ET-mediated vasoconstriction via ET A receptors [61,63,64]. In this study, the expression levels of ET A and ET B receptors were higher in mice with PTS than in mice with TTS, which may have contributed to the vessel constriction and blood flow reduction observed in the former group.

In contrast to the vasoconstriction factors, mice with TTS expressed higher levels of genes involved in vasodilation as compared to mice with PTS (Figure 7). VEGF is a potent angiogenic factor that induces endothelial cell proliferation, promotes cell migration, and inhibits apoptosis [65,66]. Modiolus, spiral ganglion, spiral ligament, supporting cells, and stria vascularis produce VEGF, and expression levels of cochlear VEGF are increased in noise-exposed mice, whereas VEGF receptor expression levels do not change [65,67]. Vlajkovic et al. observed increased expression of ADORA2A in the cochlea after noise exposure and this gene is involved in vasodilation. The authors postulated that ADORA2A suppresses expression of proinflammatory mediators via the PI3K-PKA-Akt-GSK-3β-NF-κB pathway, which contributes to the repair of cochlear tissue damage [68]. In our study, increased AT2, eNOS, and VEGF-A levels were observed in mice with TT as compared with normal controls (Figure 7e,f,h). Other “vasodilating” genes, such as ADORA2A and PGE2, were increased in mice with TTS as compared to mice with PTS (Figure 7g,i). Overall, these data indicate that vasodilating genes are expressed at significantly higher levels in TTS than PTS mice.

Our gene expression profile can be explained as follows: the genetic messages involved in vasoconstriction and dilation contribute to the overall physiological phenotypes of inner ear vasculature and blood flow, instead of contributing to each as a single factor. It seems clear that the cochlear tissues in mice with TTS can produce “stop-constriction” or “promote-dilation” messages, as indicated by the reduced expression of vasoconstrictive genes and increased expression of vasodilating genes. In contrast, the cochlear tissues of PTS mice failed to generate those messages, potentially because the fibrocytes or pericytes in the cochlea (i.e., the sources of those messages) underwent severe cell death after PTS. The capillary networks of the cochlear lateral wall contain a rich population of pericytes [69,70]; these cells are generally located on microvessels, including arterioles and venules [71]. The cochlear capillary system has a relatively large population of pericytes. The ratio of pericytes to endothelial cells is approximately 1:2 in the stria vascularis and spiral ligament, whereas the ratios are 1:1 in the retina, 1:5 in the brain, 1:10 in the lung, and 1:100 in skeletal muscle [69,72,73]. Pericytes on the blood vessels of the spiral ligament produce contractile proteins, including α-SMA, desmin, F-actin, and tropomyosin, which modulate vasocontractility [69,74]. Taken together, these data indicate that modulation of vascular tone is orchestrated by a strict balance between vasoconstriction and vasodilation factors in the cochlear lateral wall.

Reduction of blood flow in various organs induces cellular damage by producing reactive oxygen species (ROS) and releasing inflammatory cytokines [75,76,77,78]. In our study, mice with PTS exhibited significantly higher levels of antioxidant enzymatic scavengers such as catalase and HO-1 as compared to mice with TTS (Figure 8a,b), which indicates that PTS induces the production of excessive free radical species that require increased ROS detoxification. Noise-induced oxidative stress in cochlear tissues is well-documented [79,80,81]. Importantly, Yuan et al. reported that noise-induced oxidative responses as indicated by products of lipid oxidation (4-hydroxynonenal) and protein nitration (3-nitrotyrosine) occurred in a noise-dose-dependent manner [82]. The results of this study are consistent with the literature in demonstrating that the levels of antioxidant enzymatic scavengers, including HO-1 and catalase, are increased in the cochlea (particularly the organ of Corti), in response to intense noise stimulation [83,84]. The natural defense system managed by antioxidant enzymatic scavengers seems to be overwhelmed by ROS accumulation, before or during initiation of TTS- or PTS-related damage. Free radical species are generated in the cochlea, including outer hair cells and cochlear lateral wall, after exposure to damaging levels of noise [82,85,86]. ROS have been detected in cochlea immediately after noise exposure, however, they are also maintained in the cochlea for seven to 10 days after noise exposure [80]. This may explain our observation of long-term elevation of the antioxidant enzymatic scavengers found in mice with PTS. The formation and accumulation of ROS occurs through various mechanisms, depending on the type and intensity of stress. While the precise origin of ROS in the cochlea after noise exposure remains unknown [82], we speculate that a surge of oxidative stress may be caused by prolonged tissue hypoxia induced by a reduction in blood flow (Figure 4a) resulting from vasoconstriction (Figure 7a–d) in the cochlea after acoustic overstimulation. Other investigators have postulated that increased mitochondrial activity, ischemic conditions, and rebound hyperperfusion may contribute to ROS production [87,88].

Stress signaling, such as noise trauma, regulates the expression of inflammatory mediators [89]. Both reduced blood flow and ROS generation trigger local inflammation [89,90,91]. Noise exposure upregulates cytokines in the cochlea [92,93], accompanied by a significantly augmented ABR threshold shift [94]. We observed increased expression of proinflammatory cytokines (e.g., IL-1β and IL-6) in mice with PTS as compared to mice with TTS (Figure 8c–e). Yamamoto et al. speculated that generation of inflammatory mediators could occur through activation of NF-κB signaling cascade, causing cytokine production [95]. The tendency for cytokines, such as TNFα, to damage the cochlea are well-documented [96]. Alternatively, immune infiltration to the middle ear and auditory cortex may be a contributing factor in the immune response after noise trauma. In one study, the expression levels of *TNF-α, IL-1β*, and *ICAM1* genes were increased in response to noise damage [97]; in addition, elevated proinflammatory cytokine expression and microglial activation in the auditory cortex have been found in NIHL model [98]. It is important to recognize that, although immune response genes were detected in whole cochlear tissue homogenates after noise damage in our study, it is much more likely that only a subset of cell types in the inner ear upregulate these genes. Identifying these cell types could enable specific targeting of their contribution to lateral-wall and hair-cell damage in response to acoustic trauma. Our data suggest that the production of ROS and proinflammatory cytokines may play a key role in response to noise damage. Although our data do not directly explain which type of damage induces subsequent signaling activities, ROS reportedly can activate NF-κB, inducing the production of proinflammatory cytokines and the expression of stress and apoptotic genes [89].

In conclusion, mice with PTS exhibited significant reductions in cochlear blood flow, vessel diameter in the stria vascularis, and number of type IV fibrocytes in the spiral ligament as compared to mice with TTS; this was accompanied by reduced expression levels of genes involved in vasodilation and increased expression of genes involved in vasoconstriction. PTS mice also exhibited swelling of the cochlear lateral wall and higher expression levels of proinflammatory cytokines. These findings imply a potential mechanism underlying the effects of noise trauma on cochlear microcirculation and the lateral wall, and suggest that cochlear blood flow and fibrocytes are differentially modulated in a noise dose-dependent fashion.

## 4. Materials and Methods

### 4.1. Experimental Animals and Design

All animal experiments were approved by the Chungnam National University, Institutional Animal Care and Use Committee (IACUC CNU01169, 26/12/2018). C57BL/6 male mice (*n* = 76), aged 6 weeks, weighing 20 to 30 g, were used in this study after confirming to have normal hearing prior to noise exposure. Animals were randomly assigned to one of two groups according to the noise exposure level, a transient threshold shift (TTS) group, and a permanent threshold shift (PTS) group. The experimental animals were used for time point studies at immediately after (0 h) and days 1, 3, 7, and 14 following noise exposure. All experimental protocols were approved by the Chungnam National University Institutional Animal Care and Use Committee. All animal care and use was conducted in accordance with the Guide for the Care and Use of Laboratory Animals.

### 4.2. Noise Exposure

Noise exposure was induced as described previously [48,99]. Briefly, in the TTS group, animals were exposed to free-field broadband noise (1–8 kHz) for 5 min at an intensity of 108 decibels (dB) SPL. In the PTS group, animals were applied to free-field broadband noise (2–8 kHz) for 2 h at an intensity of 116 dB SPL in an acoustically insulated reverberation chamber. The noise signals were sent through an amplifier (INTER-M R300 Plus power amplifier; Canford Audio PLC, Washington, UK) and a computer to a loud speaker (ElectroVoice DH1A-WP; Sonic Electronix Inc., Los Angeles, CA, USA). The noise level was measured using a sound level meter (B & K type 2250; Brüel & Kjaer, Copenhagen, Denmark), sound calibrator (B & K type 4231; Brüel & Kjaer), and condenser microphone (B & K type 4189; Brüel & Kjaer).

### 4.3. Auditory Brainstem Response

To measure ABR thresholds at frequencies between 4 and 32 kHz, and click sounds separately from both ears, mice were anesthetized with intramuscular injection of xylazine 10 mg/kg (Rompun, Bayer Animal Health, Monheim, Germany) and zolazepam HCl 40 mg/kg (Zoletil, Virbac Animal Health, Carros, France) [99]. ABR thresholds were obtained prior to noise exposure, immediately after (0 h), and 1, 3, and 7 days after TTS or PTS using the TDT System-3 (Tucker Davis Technologies, Gainesville, FL, USA) hardware and software, as described previously [100]. Briefly, subcutaneous needle electrodes were placed around both infra-auricular areas and the skull vertex. The waveforms were analyzed by BioSig RP (version 4.4.1; Tucker Davis Technologies) and threshold was defined as the lowest stimulus intensity to evoke a wave III response > 0.2 μV.

### 4.4. Hematoxylin Staining

Hematoxylin staining was used to visualize nucleic acids of the cells in the cochlea and measure stria vascularis thickness [99,101]. Harvested tissue samples were placed in 4% paraformaldehyde in PBS for 24 h, at 4 °C, decalcified in 10% EDTA for 1 week at room temperature, embedded in paraffin, sectioned on a mechanical implant microtome (Leica RM2235, Leica Microsystems, Wetzlar, Germany) at a thickness of 4 μm, and stained with hematoxylin (Sigma-Aldrich, St. Louis, MO, USA). The stained tissue sections were photographed using a slide scanner (Panoramic MIDI version 1.23, 3DHISTECH, Ltd., Budapest, Hungary) and the numbers of hematoxylin-positive cells were quantified.

### 4.5. Transmission Electron Microscope (TEM)

Tissue samples were fixed with 3% glutaraldehyde in culture medium for 2 h at room temperature. They were washed five times with 0.1 M cacodylate buffer containing 0.1% CaCl_2_ at 4 °C. Then, they were postfixed with 1% OsO 4 in 0.1 M cacodylate buffer (pH 7.2) containing 0.1% CaCl_2_ for 2 h, at 4 °C. After rinsing with cold distilled water, tissue samples were dehydrated slowly with an ethanol series and propylene oxide at 4 °C. The cells were embedded in Spurr’s epoxy resin [102]. After polymerization of the resin at 70 °C for 36 h, serial sections were cut with a diamond knife on an ULTRACUT UCT ultramicrotome (Leica Mikrosysteme GmbH, Vienna, Austria) and mounted on formvar-coated slot grids. Sections were stained with 4% uranyl acetate for 10 min and lead citrate [103] for 7 min. They were observed by a Tecnai G2 Spirit Twin transmission electron microscope (FEI Company, Hillsboro, OR, USA).

### 4.6. Measurement of Cochlear Blood Flow

To evaluate changes in cochlear blood flow before and after noise exposure, the left tympanic bulla of each mouse was exposed and opened under anesthesia. After the mouse was placed on the stereotaxic instrument, a 0.1 mm diameter laser Doppler probe was placed over the lateral wall of the cochlea. Cochlear blood flow was determined from an intensity oscillation that was translated from the oscillation frequency produced by the Doppler frequency shift of the RBC in the left tympanic bulla, using a Laser Doppler Flowmeter (Transonic Systems, Ithaca, NY, USA). Each intensity oscillation was measured separately, and relative cochlear blood flow was reported as the ratio of the control (pre) value to the post-noise exposure value.

### 4.7. Measurement of the Stria Capillary Thickness

The cochlear lateral wall tissues were collected and fixed in 4% paraformaldehyde in PBS for 1 h at room temperature. Tissues were permeated and blocked with 0.3% Triton X-100/10% normal goat serum/PBS for 1 h and then incubated with PECAM1 (Millipore, Burlington, MA, USA), MAB 13982, IgG) antibody at a concentration of 1:200 in blocking solution overnight at 4 °C. After rinsing six times in PBS for 10 min, Alexa Fluor 594 goat anti rat IgG (A11007, 1:200, Thermo Fisher Scientific, Waltham, MA USA) was used as a secondary antibody for PECAM. The specimens were mounted on glass slides using 50% glycerol and observed using a confocal microscopy TCS SP8 (Leica Microsystems, Wetzlar, Germany). The thickness of the stria vascularis was measured, as described previously [104], using image analysis software (Case Viewer, version 2.1, 3DHISTECH, Ltd. Budapest, Hungary).

### 4.8. Quantitative Real-Time Polymerase Chain Reaction (qRT-PCR)

Quantitative RT-PCR was performed as previously described [48,99]. Briefly, tissues were collected and frozen immediately in liquid nitrogen. Total RNA was extracted with TRIzol reagent (Thermo Fisher Scientific, Waltham, MA USA). RNA was quantified using a Nano drop (Nano Drop Technologies, Wilmington, DE, USA). The cDNA was produced using the cDNA synthesis kit (Roche, Branchburg, NJ, USA). Real-time PCR was performed on a CFX Connect Real-Time PCR Detection System (BioRad, Des Plaines, IL, USA) by using a reaction mixture with SYBR Green as the fluorescent dye (Applied Biosystems, Waltham, Massachusetts, USA). The fold change (2^−Δ(ΔC^_T_^)^) in the target gene relative to the endogenous control gene was calculated.

### 4.9. Image Processing and Statistical Analysis

Adobe Photoshop (version 7.0) was used for adjustment of image contrast, superimposition of images, and colorization of monochrome fluorescence images. A two-way ANOVA coded for treatment (TTS and PTS) and day was used for ABR, cochlea blood flow, and qRT-PCR. For vessel thickness in the stria vascularis, a one-way ANOVA was used. An unpaired Student’s t-test was used for all other comparisons. A *p*-value < 0.05 was significant in each case. All tests were performed using GraphPad Prism (Version 6).

## Abbreviations

TTS	transient hearing threshold shift
PTS	permanent hearing threshold shift
IL	interleukin
TNF	tumor necrosis factor
NIHL	noise-induced hearing loss
SLFs	spiral ligament fibrocytes
SGNs	spiral ganglion neurons
ADRA1A	alpha-1A adrenergic receptor
ADRA1D	alpha-1D adrenergic receptor
ET A	endothelin receptor type A
ET B	endothelin receptor type B
HO-1	heme oxygenase 1
eNOS	endothelial nitric oxide synthase
AT2	angiotensin 2 receptor type 2
ADORA2A	adenosine A2A receptor
PGE2	prostaglandin E receptor 2
PGI2	prostaglandin I2 receptor
VEGF-A	vascular endothelial growth factor A
